# Simethicone Medication Should Be Avoided in Infants Receiving Oral Lactase Treatment

**DOI:** 10.3390/children11081009

**Published:** 2024-08-17

**Authors:** Ekin Say Yildirim, Adem Aydin, Tolga Ince, Zeynep Varol, Belgin Ünal, And Demir

**Affiliations:** 1 Department of Pediatrics, Faculty of Medicine, Dokuz Eylül University, 35340 İzmir, Türkiye; 2 Department of Public Health, Faculty of Medicine, Dokuz Eylül University, 35340 İzmir, Türkiye; 3 Pediatric Research Center, New Children’s Hospital, University of Helsinki, Helsinki University Hospital, 00290 Helsinki, Finland

**Keywords:** lactose intolerance, infant, lactase, simethicone

## Abstract

Objective: In this retrospective study, we assessed the efficacy of oral lactase in infants with lactose intolerance in terms of sex, birth weight, and interference by other medications. Methods: The study was conducted on patients aged 0–6 months who had been diagnosed with lactose intolerance on admission to the Department of Social Pediatrics at Dokuz Eylul University. Demographic data, the onset of symptoms, and medications used were recorded. Results: We found that 86.7% of the infants responded to lactase, with no significant differences based on sex, birth weight, or age at symptom presentation. We observed, however, that the efficacy of treatment did improve over time, thereby deriving benefits from intestinal development and adaptation. Interestingly, the response to lactase was reduced in infants receiving concomitant simethicone for colic symptoms, suggesting a potential drug interaction, while probiotics had no impact on treatment outcomes. Conclusions: We advise against using additional medications with oral lactase, pointing out a possible interaction with simethicone that might decrease the effectiveness of treatment.

## 1. Introduction

The pathophysiology of lactase deficiency involves lactose intolerance due to the non-absorption of lactose, which presents itself with gastrointestinal symptoms [1]. The severity of these symptoms, mainly abdominal pain, bloating, diarrhea, and occasional vomiting seen in infants, correlates with the amount of lactose ingested; however, not with the degree of lactase deficiency [2]. These symptoms can easily be misdiagnosed as infantile colic because they occur in the same age range. Primary lactase deficiency is quite widespread, with a prevalence of 60–80% worldwide. On the other hand, secondary lactase deficiency develops as a result of infections or diseases that damage the intestines, such as celiac disease. Infants born with a rare, congenital lactase deficiency suffer severe symptoms following lactose intake. Diagnostic techniques include hydrogen breath tests, stool-acidity tests, reducing-substances tests, and intestinal biopsies, while treatment includes lactose-reducing formulas with additional supplements to relieve symptoms [3].

Treatment of lactase intolerance consists mainly of symptomatic dietary modifications, with supplementation of the lactase enzyme [4]. Dairy avoidance is not recommended given the necessity of calcium and vitamin D for bone health. On the other hand, lactose-free cow’s milk protein-based formulas do not seem to positively affect infant growth and development or colic symptoms [5]. Oral lactase replacement capsules or the use of lactase-enriched milk products allow these patients to enjoy dairy products [6]. Oral lactase supplementation is effective in adults and children for relieving symptoms. Finally, many infants are placed on simethicone for colic, but multiple studies have shown this medication to be no more effective than a placebo [7,8]. There is currently no clear consensus in the medical literature regarding the management of infantile colic [9]. Persistent or excessive crying (colic) is one of the most distressing problems of infancy. Consequently, parents also feel obliged to seek alternative solutions simultaneously, and the use of unprescribed simethicone or probiotics containing *Lactobacillus reuteri* for the treatment of colic symptoms is widespread in infants receiving oral lactase treatment.

In this study, we examined the impact of oral lactase therapy on 0–6-month-old breastfed neonates with positive stool reductant tests and low stool pH, using stratification by sex, birth weight, age when first symptoms suggestive of lactose intolerance were noticed, and interference from medications.

## 2. Materials and Methods

The study was designed as a retrospective cohort study and was approved by the Dokuz Eylül University Faculty of Medicine Non-Interventional Clinical Research Ethics Committee on 3 November 2021, decision number 2021/31-19. The study included patients who were admitted to the Department of Social Pediatrics on account of primary lactose intolerance and treated with lactase enzyme (at a dose of 8 × 390 IU) during the period from January 2018 to September 2021. Patients with secondary causes of lactose intolerance were excluded from the study. The patient population was composed of 113 exclusively breastfed infants with lactose intolerance, aged 0–6 months. Lactose intolerance was investigated and diagnosed by employing a positive stool reducing-substances test and a confirming stool-acidity test in infants with one or more complaints of abdominal pain, diarrhea, nausea, gas, and/or bloating after lactose or lactose-containing food intake. Data collection was carried out by filling in a pre-designed questionnaire answered by the parents or caregivers, who were contacted by email or phone.

Fresh stool samples, stored for no more than one hour, were collected at a private laboratory setting in Izmir, Türkiye. These samples underwent a stool reducing-substances test involving mixing and boiling 2 milliliters of Benedict’s solution until there was a color change, as unabsorbed sugars reduce copper ions in Benedict’s solution. After boiling, samples were centrifuged for five minutes; then the test result was confirmed as positive when the supernatant changed to a greenish-brown color. Additionally, as a measure of confirmation, stool acidity was determined using pH paper.

Data on sex, week of birth, birth weight, time of intestinal complaint onset, frequency and duration of the intestinal complaint, correlation of these complaints with breast milk intake, stool characteristics, extent and duration of lactase enzyme use, administration of other medicines, and other complaints like rash or redness were recorded. The effectiveness of oral lactase treatment given before breastfeeding in reducing intestinal complaints was also recorded based on the mother’s observation.

All statistical analyses were performed using SPSS (version 20.0). Descriptive data are presented as frequencies for categorical variables and means and standard deviations for continuous variables. The chi-square test was used to analyze associations between demographic and clinical characteristics and benefiting from lactase enzyme treatment. A *p-*value of < 0.05 was considered statistically significant.

## 3. Results

### 3.1. Demographic Findings

Table 1 provides the sociodemographic and clinical characteristics of the infants in the study, which are essential for understanding the baseline characteristics of the study population.

### 3.2. Intestinal Symptoms and Complaints

Table 2 summarizes the symptoms and complaints reported by the study group and details the specific symptoms experienced; the occurrence of vomiting; and the timing, duration, and frequency of intestinal issues. Intestinal problems began at birth in 19.5% of children, at 2–3 weeks in 37.2%, at 1–3 months in 36.3%, and at 3–6 months in 5.3%. The exact symptoms experienced included abdominal distension only in 6.2%, restlessness in 30.1%, and both symptoms in 58.4% of the cases. Regarding the timing of these complaints, 49.6% of the children experienced them at any time of the day, while 41.4% experienced them later in the evening. Concerning frequency, 13.3% of cases experienced complaints less than three days a week, and 77.9% had complaints more than three days a week. Additionally, there were duration-wise differences in the complaints: 23.9% had them for fewer than 3 h a day, and 56.6% had them for more than 3 h a day. Vomiting was observed in 31.9% of the cases (Table 2).

### 3.3. The Characteristics of Stool at the Onset of Symptoms

In the study group, 30.1% of infants had stools with blood streaks, 1.8% had bloody and watery stools, 5.3% had stools that were between solid and liquid with blood, 56.6% had stools that were between solid and liquid without blood, and 6.2% had solid stools. Additionally, 25.7% passed stool fewer than three times a day, 51.3% passed stool more than three times a day, 8% defecated every two to three days, and 5.3% once a week following the manifestation of intestinal complaints. In 39.8% of the infants, complaints presented within one hour after breastfeeding, and another 25.7% exhibited corresponding symptoms more than one hour after breastfeeding. Intestinal complaints increased in 44.2% of infants upon breast milk intake but decreased in another 31% of the infants. Atopic dermatitis was seen in 4.4% of the infants, facial rash in 2.7%, and 8.8% had both, whereas 84% had no additional complaints.

### 3.4. Stool Reducing Substances and Stool pH in Infants

Results on stool reducing-substances positivity levels and stool pH are presented in Table 3. The results indicate that 69.1% of infants had a positivity level of the stool reducing substances at two or below, while 30.1% had a level of three or higher. Additionally, 74.3% of the infants had a stool pH of less than 7, and 23.9% had a pH of 7 or above (Table 3).

### 3.5. Information on the Use of Lactase Enzymes

The ages at which infants started using the lactase enzyme ranged from 8 to 179 days, with a mean starting age of 52.58 ± 32.99 days. The duration of lactase enzyme use varied from 30 to 450 days, with a mean of 137.60 ± 95.20 days.

The other medication that was prescribed for the infants was probiotics containing *Lactobacillus reuteri*, used by 23% of the participants, and simethicone drops, used by 20.3%. All the infants were given lactase enzyme and 86.7% of the infants benefited from the use of lactase, while 13.3% did not (Table 4).

### 3.6. Demographic Data and Clinical Characteristics Related to the Benefit from Lactase Enzyme Use

Data referring to the sex and intestinal complaints of infants who benefited or did not benefit from using lactase enzyme are presented in Table 5. As observed in Table 5, 86.0% of males and 87.5% of females benefitted from lactase use, and there was no statistically significant difference between males and females (*p* = 0.81).

Among the 64 children whose complaints started before one month of age, 87.5% benefited from lactase enzyme use compared with 85% of the 49 children whose complaints started at or after one month of age. There was no statistically significant association between the month of onset of complaints and benefits from the lactase enzyme (*p* = 0.78).

The children with symptoms of both abdominal distension and restlessness (89.3%) and those with one symptom alone (83.0%) did not show any statistically significant difference regarding the benefit from the lactase enzyme use (*p* = 0.32).

There was a statistically significant difference regarding the benefit of lactase enzyme use between children with complaints at any time of the day (92.8%) and those with complaints later in the day (79.6%) (*p* = 0.04).

Among the 98 children with complaints more than three days per week, 76.5% benefited from lactase enzyme. Among the 15 children with fewer than three days of complaints per week, 86.6% benefited (*p* = 0.51). There was no statistically significant association between stool characteristics, defecation frequency, and benefits from the lactase enzyme (*p =* 0.81 and *p* = 0.12, respectively).

### 3.7. Intake of Breast Milk and Benefit from Lactase Enzyme

Among the 45 infants who experienced intestinal complaints within the first hour after breastfeeding, 84.4% showed improvement with the administration of the lactase enzyme. Similarly, among the 82 infants whose intestinal complaints began more than one hour after breastfeeding, 86.5% benefited from the enzyme. There was no statistically significant association between the timing of complaints after breastfeeding and the benefit from oral lactase treatment (*p =* 0.89). There was no statistically significant difference in benefitting from lactase enzyme use between infants whose complaints increased with breastfeeding (84%) and infants whose complaints did not increase with breast milk intake (82.8%) (*p =* 0.32).

### 3.8. Other Complaints and Lactase Enzyme Benefit

Among the 18 infants with other additional complaints, 88.8% benefited from the enzyme use, and among the 95 infants with no other complaints, 86.3% benefited. No significant association was noted between the presence of different complaints and the benefits from the lactase enzyme (*p =* 1.00).

### 3.9. Concurrent Use of Unprescribed Medication and Its Effect on Benefit from Lactase Treatment

The relationship between the concurrent use of unprescribed medication and the benefit from lactase treatment is shown in Table 6. Among the 22 infants who were taking medications containing simethicone, 59% benefited from the enzyme; in contrast, of 91 infants not using simethicone-containing drugs, 93.4% benefited. A significantly low response to lactase treatment was noted in the children who were concurrently given simethicone-containing drugs compared with those getting only oral lactase treatment (*p* = 0.02). Among the 87 children given probiotics containing the *Lactobacillus reuteri* strain, 86.2% benefited from the enzyme, and among 26 children who did not use probiotics, 88.4% benefited. No statistically significant difference in benefit from lactase use was found between subjects who were taking probiotics concurrently and those not taking them (*p* = 0.32).

## 4. Discussion

Our findings support that oral lactase treatment is effective in infants with lactose intolerance, a potential cause of infantile colic, particularly in those 0–6 months old and exclusively breastfed. It is advisable to continue oral lactase administration without the concurrent use of any additional medications, as the efficacy of lactase treatment tends to increase over time, potentially due to intestinal development and adaptation.

Almost nine out of ten patients in our cohort benefited from oral lactase treatment. This finding aligns with several studies in adults with lactose intolerance, where this oral lactase enzyme was found to be effective. Notable among them are the studies by Ibba et al. and Ojetti et al., which employed clinical, genetic, and hydrogen breath tests for diagnosis [10,11]. Montalto et al. arrived at similar findings in their study of 30 adult patients. However, very few studies have been conducted on infants and children due to the difficulty in diagnosing lactose intolerance and the varied responses to treatment in this age group [12]. A study by Wang et al. on preterm infants reported that oral lactase treatment significantly improved clinical symptoms and reduced substances in stools with stable pH values [13].

Regarding the relationship between lactose intolerance and birth weight, Erasmus et al. reported improved weight gain with oral lactase treatment in 130 premature infants [14]. The mean birth weight of lactose-intolerant infants in our study was 3016 g, and we did not find any significant difference in response to oral lactase treatment based on birth weight. This could be due to the fact that we had only one low-birth-weight baby in our study. Also, we observed an equal representation of both female and male infants; hence, there was no sex difference regarding the response to treatment with oral lactase replacement. Additionally, the timing of the onset of symptoms was not associated with the response to oral lactase treatment.

Regarding symptoms, nearly two-thirds of the lactose-intolerant infants demonstrated abdominal distension and restlessness, and about one-third had one or the other. No improvement in the efficacy of the lactase enzyme could be established based on these symptoms. However, there have been reports of a marked reduction in crying time when using the lactase enzyme before feeding [15,16,17]. In contrast, Miller et al. reported no effect on crying when given post-feeding. In our research, the enzyme was administered before breastfeeding. This aligns with reports that the administration of lactase drops added to milk formula or given before breastfeeding significantly reduced crying in infants with colic [3].

In contrast, a study by Liebman et al. of 56 infants with colic showed no correlation between symptoms and stool pH or stool reducing-substances test results [18]. However, all our study patients had stool reducing-substances test scores ranging from 1+ to 5+, and 86.7% of them benefited from lactase, suggesting that the positivity of the stool reducing-substances test supports the diagnosis of lactose intolerance. No significant correlation could be found among stool pH, stool reducing-agent test score, and lactase treatment effectiveness in our cohort.

Various factors have been investigated in the literature to determine the etiology of lactose intolerance. Lactose intolerance is more frequent in infants born before 37 weeks of gestation. Our study showed that the mean gestational age was 37.9 weeks for infants diagnosed with lactose intolerance. We found no difference between preterm and full-term babies in response to the lactase enzyme. This, in turn, implies that while there is an increased prevalence of lactose intolerance in infants born earlier, oral treatment with lactase is equally effective regardless of gestational age.

A substantial proportion of infants with liquid, bloody, or solid stools benefited from the lactase enzyme, though the percentage was slightly lower than for those without blood in their stools. The *p*-value indicated no significant statistical association between stool characteristics (solid-to-liquid without blood vs. liquid, bloody, or solid stools), and the benefits from the lactase enzyme suggests that the enzyme’s effectiveness is not influenced significantly by the presence or absence of blood in the stool. Infants with a higher frequency of stools (more than three times daily) showed a higher response rate to the lactase enzyme, indicating the enzyme’s potential efficacy in frequent stool passers. The benefit rate for infants passing stool less frequently was lower compared with those with higher stool frequency but still indicated that a majority experienced positive effects from the enzyme. The *p*-value indicating no significant statistical difference between stool frequency and the benefits of oral lactase treatment suggests that the frequency of stool passing does not significantly affect the efficacy of the lactase enzyme.

Indeed, this intermittent complaint regarding lactose intolerance in infants can appear anytime throughout the day. In our study, nearly half of the subjects showed symptoms at any time of the day, with no difference in the timing of the response to the lactase enzyme. Some infants with colic whose symptoms typically worsen later in the day, have also been found to have lactose intolerance [19]. In our study, nearly four out of five infants had symptoms more than three days per week, and again, no significant difference in lactase effectiveness was found based on symptom duration.

Probiotics containing *Lactobacillus reuteri* were given to about a quarter of the patients and simethicone-containing drugs to almost a fifth. While *Lactobacillus reuteri* has been effective in various gastrointestinal issues, no significant difference in lactase effectiveness was observed in our patients using *Lactobacillus reuteri* probiotics. In contrast, infants receiving simethicone with lactase showed less significant improvement, suggesting a potential interaction between the two treatments.

In our study, around 15% of the patients presented with atopic manifestations such as rash and redness. Milk protein allergy is prevalent mainly during the first six months of life. It shares some clinical similarities with lactose intolerance but may also present with cutaneous lesions, rashes, and respiratory symptoms, none of which are features of lactose intolerance. On the other hand, patients with atopic manifestations could not be differentiated from those without additional symptoms based on the effect of the lactase enzyme. This may indicate the possibility of concurrent lactose intolerance and cow milk protein allergy and the benefit of oral treatment with lactase in these cases.

One major limitation of the study was the absence of a traditional control group. It was not feasible to establish a control group consisting of individuals with lactose intolerance who were not administered lactase treatment, as it would be unethical to diagnose the condition and then withhold treatment. Instead, within the group of participants with lactose intolerance, those who did not receive simethicone effectively served as our control group. Another limitation was the nonspecificity of the tests used (stool reducing-substances test and stool-acidity test), which are known to produce false positives. This occurs because other sugars in addition to lactose, such as fructose and sucrose, can also yield positive results if malabsorbed, potentially leading to misinterpretation. Over 200 different types of human milk oligosaccharides (HMOs), primarily glucose, galactose, N-acetylglucosamine, fucose, and sialic acid, have been identified, showing significant variation in structure and composition among different mothers and throughout the course of lactation. Additionally, mild gastrointestinal infections and rapid gastric transit, the latter typical in breast-fed infants, may cause false-positive test results. More specific tests for lactose intolerance, such as the lactose-tolerance test and hydrogen breath test, were not applicable for infants aged 0 to 6 months. Consequently, we included patients in the study if they tested positive on both the stool reducing-substances test and stool-acidity test, thereby improving the specificity rate. Notably, lactase treatment was effective in over 90% of the subjects diagnosed with lactose intolerance.

Oral lactase enzymes have been found to be very effective in reducing crying in infants with colic. The exact cause of infantile colic is still unclear, but studies indicate that approximately 40% of infants with colic may have lactase enzyme deficiency [20,21]. Considering the overlap in symptoms of colic and lactose intolerance, further research into the use of lactase for colicky infants is highly likely to be valuable. Such research could significantly contribute to devising favorable health interventions or improving psychological well-being for both the infant and the caregiver. The statistically significantly lower response to oral lactase treatment in infants receiving medications containing simethicone should be taken into account.

Most of the infants benefited from the lactase enzyme treatment across all categories. The statistically significant difference was only observed in the timing of peak complaints; the infants with complaints at all hours of the day benefited more from the treatment compared with those with later-day complaints. Additionally, there was evidence of an amplified benefit from lactase with more prolonged use, possibly due to intestinal adaptation. This supports evidence from Briet et al. that continued lactose consumption in adults might be associated with a reduction in symptoms due to changes in intestinal flora and function [22].

Overall, the concurrent use of any medication that could confound treatment outcomes, particularly simethicone, should be avoided in children undergoing oral lactase treatment. However, if additional medication is to be considered, probiotics containing the *Lactobacillus reuteri* strain may be preferred over simethicone to avoid any reduction in the effectiveness of oral lactase treatment. This insight into how simethicone can interact with the efficacy of oral lactase treatment when used to help with infantile gas and bloating possibly represents an unreported drug interaction in the treatment of lactose intolerance in infants. We conclude that this novel finding is crucial for clinical practice, underscoring the need to consider medication interactions when managing lactose intolerance in infants.

## Figures and Tables

**Table 1:** Sex, birth week, birth weight, and known disease history of the patients.

Variable	Category	Number (n)	Percentage (%)	Additional Information
Sex	Male	56	49.6	
	Female	57	50.4	
Birth week				Average: 37.9 ± 0.8
				Range: 33–41 weeks
Birth weight (g)				Average: 3026.1 ± 241.7
				Range: 1500–4100
Stool pH				5.9 ± 0.9 Range: 5–8
Stool reducing substances				2.1 ± 1.0 Range: 1–5
Identified disease	None	105	92.8	
	Multiple food allergy	2	1.8	
	Atopic dermatitis	1	0.9	
	Pollen allergy	1	0.9	
	Pulmonary stenosis	1	0.9	
	Beta thalassemia carrier	1	0.9	
	Osteogenesis imperfecta	1	0.9	
	Vision problem	1	0.9	
	Total	113	100	

**Table 2:** Characteristics of intestinal symptoms and complaints among the 113 infants.

Characteristic	Category	Percentage (%)
Exact symptom experienced	Abdominal distension only	6.2
	Restlessness	30.1
	Both symptoms	58.4
Vomiting	Observed	31.9
Onset of intestinal problems	From day one	19.5
	After 2–3 weeks	37.2
	After 1–3 months	36.3
	After 3–6 months	5.3
Timing of complaints	Any time of the day	49.6
	Later in the day	41.4
Duration of complaints	Less than 3 h a day	23.9
	More than 3 h a day	56.6
Frequency of complaints	Less than 3 days a week	13.3
	More than 3 days a week	77.9

**Table 3:** Stool reducing substances and stool pH in infants.

Parameter	Category	Number of Infants (n)	Percentage (%)
Stool reducing substances	<3	78	69.1
	≥3	34	30.1
Stool pH	pH < 7	84	74.3
	pH ≥ 7	27	23.9

**Table 4:** Age, duration, and supplementary medication used with lactase enzyme.

Parameter	Statistics	Details
Age at lactase initiation (days)	X ± SD: 52.58 ± 32.99	Range: 8–179
Duration of lactase enzyme use (days)	X ± SD: 137.60 ± 95.20	Range: 30–450
Supplementary medicine used with lactase enzyme		
- Prebiotic (*Lactobacillus reuteri*)	*n* = 26	Percentage: 23.0%
- Simethicone	*n* = 23	Percentage: 20.3%
- No additional medication	*n* = 64	Percentage: 56.7%
Benefit from the lactase enzyme use		
- Benefit	*n* = 98	Percentage: 86.7%
- No benefit	*n* = 15	Percentage: 13.3%

**Table 5:** Association between demographic and clinical characteristics of lactase enzyme efficacy in infants.

Characteristic	Category	Benefiting from Lactase Enzyme (%)	Not Benefiting from Lactase Enzyme (%)	*p*-Value
Sex	Male	49 (86.0%)	8 (14.0%)	0.81
	Female	49 (87.5%)	7 (12.5%)	
Time of first symptom occurrence	Under 1 month	56 (87.5%)	8 (12.5%)	0.78
	Over 1 month	42 (85.0%)	7 (15.0%)	
Abdominal swelling and restlessness	Having both complaints	59 (89.3%)	7 (10.7%)	0.32
	Having only one complaint	39 (83.0%)	8 (17.0%)	
Peak complaint time	At all hours of the day	52 (92.8%)	4 (6.2%)	0.04
	Later in the day	43 (79.6%)	11 (20.4%)	
Weekly frequency of the complaints	More than 3 days a week	75 (76.5%)	23 (23.5%)	0.52
	Less than 3 days a week	13 (86.6%)	2 (13.4%)	
Stool characteristics	Solid to liquid, bloodless	62 (91.0%)	6 (9.0%)	0.81
	Liquid/bloody/very solid	36 (80.0%)	9 (20.0%)	
Defecation frequency	More than 3 times a day	65 (89.0%)	8 (11.0%)	0.12
	Less than 3 times a day	22 (75.8%)	7 (24.2%)	

**Table 6:** Impact of concurrent unprescribed medication usage on the efficacy of oral lactase treatment in infants.

Medication Type	Usage Category	Benefiting from Lactase Enzyme (%)	Not Benefiting from Lactase Enzyme (%)	*p*-Value
Simethicone	Using	13 (59.0%)	9 (41.0%)	0.02
	Not using	85 (93.4%)	6 (6.6%)	
Probiotics (*Lactobacillus reuteri*)	Using	75 (86.2%)	12 (13.8%)	0.32
	Not using	23 (88.4%)	3 (11.6%)	

## Data Availability

The data presented in this study are available on request from the corresponding author. The data are not publicly available due to privacy or ethical restrictions.
